# Mobile Health Crowdsensing (MHCS) Intervention on Chronic Disease Awareness: Protocol for a Systematic Review

**DOI:** 10.2196/24589

**Published:** 2021-03-19

**Authors:** Temitope Oluwaseyi Tokosi, Michael Twum-Darko

**Affiliations:** 1 Graduate Centre for Management Faculty of Business and Management Sciences Cape Peninsula University of Technology Cape Town South Africa

**Keywords:** mHealth, crowdsensing, chronic diseases, awareness, mobile phone

## Abstract

**Background:**

Mobile health crowdsensing (MHCS) involves the use of mobile communication technologies to promote health by supporting health care practices (eg, health data collection, delivery of health care information, or patient observation and provision of care). MHCS technologies (eg, smartphones) have sensory capabilities, such as GPS, voice, light, and camera, to collect, analyze, and share user-centered data (explicit and implicit). The current literature indicates no scientific study related to MHCS interventions for chronic diseases. The proposed systematic review will examine the impact of MHCS interventions on chronic disease awareness.

**Objective:**

The objectives of this study are to identify and describe various MHCS intervention strategies applied to chronic disease awareness.

**Methods:**

Literature from various databases, such as MEDLINE, Embase, PsycINFO, CINAHL, and Cochrane Central Register of Controlled Trials, will be examined. Trial registers, reports, grey literature, and unpublished academic theses will also be included. All mobile technologies, such as cell phones, personal digital assistants, and tablets that have short message service, multimedia message service, video, and audio capabilities, will be included. MHCS will be the primary intervention strategy. The search strategy will include keywords such as *mHealth*, *crowdsensing*, and *awareness* among other medical subject heading terms. Articles published from January 1, 1945, to December 31, 2019, will be eligible for inclusion. The authors will independently screen and select studies, extract data, and assess the risk of bias, with discrepancies resolved by an independent party not involved in the study. The authors will assess statistical heterogeneity by examining the types of participants, interventions, study designs, and outcomes in each study, and pool studies judged to be statistically homogeneous. In the assessment of heterogeneity, a sensitivity analysis will be considered to explore statistical heterogeneity. Statistical heterogeneity will be investigated using the chi-square test of homogeneity on Cochrane *Q* test, and quantified using the *I*^2^ statistic.

**Results:**

The preliminary search query found 1 paper**.** Further literature search commenced in mid-March 2021 and is to be concluded in April 2021. The proposed systematic review protocol has been registered in PROSPERO (The International Prospective Register of Systematic Reviews; no. CRD42020161435). Furthermore, the use of search data extraction and capturing in Review Manager version 5.3 (Cochrane) commenced in January 2021 and ended in February 2021. Further literature search will begin in mid-March 2021 and will be concluded in April 2021. The final stages will include analyses and writing, which are anticipated to start and be completed in May 2021.

**Conclusions:**

The knowledge derived from this study will inform health care stakeholders—including researchers, policy makers, investors, health professionals, technologists, and engineers—of the impact of MHCS interventions on chronic disease awareness.

**International Registered Report Identifier (IRRID):**

PRR1-10.2196/24589

## Introduction

Mobile health (mHealth) is a component of eHealth that involves the use of mobile communication technologies to promote health by supporting health care practices (eg, health data collection, health care information delivery, or provision of care and patient observation) [1]. Crowdsensing is a paradigm, which, as advocated by Banda [2], is based on the crowdsourcing concept of engaging a crowd to solve a complex problem through an open forum. The term *mobile* in *mobile crowdsensing* means using mobile smart devices, such as smartphones, and involving human mobility when using these devices [3]. mHealth crowdsensing (MHCS), on the other hand, takes advantage of pervasive mobile smart devices to efficiently collect data, enabling numerous large-scale applications [4]. It is made possible through human involvement, which is an important key feature.

Similarly, MHCS applications have been reported in studies by Pryss et al [5], who investigated a MHCS platform, Track Your Tinnitus. This platform uses smart mobile devices, a website, a backend system, and 2 mobile applications for patients over a 12-month period. Similarly, Giannotta [6] undertook research to design an MHCS application called Track your Diabetes. In the study by Bellavista et al [7], an MHCS-based middleware known as Collaborative Emergency Group Assistant (COLLEGA) was shown to enhance the potential of supporting participatory management of mHealth communities for emergency scenarios. Furthermore, the study of Reddy et al. [8] described DietSense, a software system that supports people who want to lose weight. At last, the study of Gao et al [9] involved designing a novel health-aware smartphone system, HealthAware, which uses an embedded accelerometer to monitor daily physical activities and a built-in camera to analyze food items.

The mobile devices or technologies applied to health include mobile phones, such as smartphones, tablets, portable media players, and their mHealth apps [10]. Smartphones, having sensory capabilities, such GPS, voice, light, and camera, are the devices most suitable for MHCS research, as they have the ability to collect, analyze, and share user-centered data (explicit and implicit) [11].

Understanding chronic disease follows Goodman et al’s [12] argument that there is no single uniform definition. For this paper, the authors combine and adopt the Centers for Disease Control and Prevention (CDC) [13] and Health and Human Services (HHS) [14] definitions, with the former defining chronic diseases as “conditions that last 1 year or more and require ongoing medical attention or limit activities of daily living or both” [13].

Chronic diseases are synonymous with infectious diseases. Tokosi et al [3] undertook a scoping review on MHCS applied to chronic diseases, including arthritis, asthma, cancer, diabetes, HIV, obesity, sclerosis, and tinnitus. COVID-19 does not fit the definition of a chronic disease for this study.

Some research cases highlighting MHCS intervention for chronic diseases are presented here. For example, the study by Edoh [15] showcased a proof of concept in the prevention of the spread of emerging infectious diseases using a hybrid crowdsensing paradigm. The study revealed positive results, such as a potential to improve conventional epidemiological data collection. Pryss et al’s [5] study highlights MHCS intervention for the chronic disease tinnitus through awareness, diagnosis, and potential treatment; meanwhile, their study on mobile crowdsensing services for tinnitus assessment and patient feedback revealed that mobile feedback service can assist patients in demystifying tinnitus and taking control of it, which should facilitate them to better cope with the condition [16].

The objectives of this study are to identify and describe the various MHCS intervention strategies used for chronic diseases and to assess their impact on chronic disease awareness. A motivation for this study is that MHCS technology has rarely been used in clinical contexts [17].

## Methods

This manuscript adheres to the PRISMA-P (Preferred Reporting Items for Systematic Reviews and Meta-Analysis Protocols) 2015 checklist (Multimedia Appendix 1) in line with a submission for systematic review protocols [18]. This protocol has been registered with PROSPERO (the International Database of Prospective Register of Systematic Reviews; no. CRD42020161435) [19].

### Study Design

This review will include randomized and nonrandomized studies. For nonrandomized studies, case–control, cohort, and cross-sectional studies in which MHCS was the main primary intervention strategy used for chronic disease awareness will be included. Studies in which MHCS was used as an intervention strategy for chronic disease screening will also be included.

### Study Participants

Study participants will be both female and male, and no age restriction will be applied. Individuals of any race, ethnicity, employment status, occupation, and geographical location will be eligible for inclusion.

### Types of Interventions

The relevant MHCS interventions for this study focus primarily on positively impacting chronic diseases awareness. MHCS interventions for health care consumers vary. For example, in the study by Pryss et al [16], MHCS was designed to provide patients with aggregated information about the variation of their tinnitus over time. This is a form of health awareness strategy where the mobile feedback service helps a patient to understand, get better control of, and cope with their chronic health condition. Meanwhile, he Free et al’s [20] strategy categorizes by device (eg, mobile phone, personal digital assistant [PDA]) and modality (eg, SMS, text messaging, multimedia message service [MMS], video). This will be used in describing the interventions [20].

### Types of Technology

Mobile devices having sensory capabilities, such as GPS, camera, and light, is a typical feature of the technology used for MHCS. Also, cellular communication that allows for wireless and 3G/4G capabilities will be part of the study. These devices include mobile phones (including Android and IOS smartphones), PDAs and PDA phones, tablets, smartphone apps, ultraportable computers, and smart books [10,21].

MHCS smartphone functionalities comprise voice over internet protocol, SMS, text messaging, MMS, GPS, Bluetooth, audio, email, light sensing, and internet [22]. Some applications using MHCS modalities include data collection (web) [23,24], patient health education (web) [6], patient health self-management (text) [25,26], behaviour change communication (images, audio, text) [27], sensors and point-of-care devices (camera, microphone, GPS, accelerometer, digital compass, Bluetooth sensing) [28], provider communication (SMS, MMS, smartphone camera), provider training and education (SMS, MMS, audio, video), human resource management (voice, SMS), supply chain management (GPS, SMS), and financial transactions and incentives (mobile banking service, airtime transfers) [10,17,29].

### Outcomes

The proposed MHCS intervention impact on chronic disease awareness will be reviewed by assessing the following: the increased attendance at clinics for chronic diseases; the stage of chronic diseases when diagnosed (as this will assist in determining whether MHCS has promoted early detection and screening); and increased chronic disease inquiry via call centres, online forums, and social media. User acceptability will not be assessed as an outcome.

Chronic disease awareness is described as the ability to be fully informed and knowledgeable of a terminal disease suffered by a patient [30]. Furthermore, where actual patient numbers cannot be determined, the baseline for assessment will be determined by the keywords *increase*, *improvement*, or, *rise* used in the study.

### Study Setting

Geographical setting will not be restrictive in the study. All available health facilities where MHCS research on chronic diseases were conducted will be included. This approach allows for all relevant information sources to be captured.

### Exclusion Criteria

Study types will be excluded according to the following criteria described by Tokosi et al [25]: non–English language papers; studies before January 1, 1999, and after December 31, 2019; nonhealth-related studies; letters, commentaries, and editorials; studies lacking primary data or explicit method description; duplicate studies that are published in more than one journal or report (the most comprehensive and up-to-date version will be used); studies not including human involvement; and studies not having a health-technology application focus.

### Search Strategy

Banda [2] highlights the origins of crowdsourcing that date back to the 19th century when Joseph Henry created the first national weather map of the United States in 1856 using a new networked technology of his day (the telegraph) to crowdsource weather reports from across the country [31]. In our study, the earliest start date will be when each database began operation. All major databases, including MEDLINE, Embase, PsycINFO, CINAHL, The Cochrane Library (Cochrane Database of Systematic Reviews, Cochrane Central Register of Controlled Trials, Cochrane Methodology Register), National Health Services Health Technology Assessment Database, Scopus, Web of Science, and Google Scholar, will be included [30]. The language of publication will be limited to English for reasonable analysis purposes.

### Other Sources

The phrase *chronic disease* will be used to expand the search strategy (where necessary), which will include grey literature and other databases, including SpringerLink, Wiley InterScience, trial registers, Institute of Electrical and Electronics Engineers, Association for Computing Machinery Digital Library, and CiteSeer [29]. We concur with the experimental findings proposed by Fortuin et al [10] in identifying accurate search terms in the development of an optimum search strategy. For trial registers, we will identify ongoing studies and recently completed trials. The studies to be included will be selected using predefined search terms adapted for the databases to be used. Full-text articles of studies extracted from reference lists will be reviewed. Manual searches of reference lists of primary studies, and of relevant and previously published reviews will be completed. Grey literature will comprise unpublished studies identified in universities and other institutional repositories, with the same eligibility criteria applied. The MEDLINE format for searching key terms is detailed in Table 1 and in Multimedia Appendix 2. The number of identified references following a preliminary search is reported in Table 1.

**Table 1:** Preliminary search query classification.

Number	Query^a^	Results, n
1	Search: ((((((((((((mhealth) OR (telemedicine)) OR (wireless technology)) OR (mobile phone)) OR (smartphone)) OR (cellphone)) OR (mobile technology)) OR (mobile device)) OR (mobile-based phone)) OR (tablet computer)) OR (IPAD)) OR (pda)) OR (mhealth application)	102,787
2	Search: (((((((((((((mhealth) OR (telemedicine)) OR (wireless technology)) OR (mobile phone)) OR (smartphone)) OR (cell phone)) OR (mobile technology)) OR (mobile device)) OR (mobile-based phone)) OR (tablet computer)) OR (IPAD)) OR (pda)) OR (mhealth application)) AND (crowdsourcing)	162
3	Search: ((((((((((((((mhealth) OR (telemedicine)) OR (wireless technology)) OR (mobile phone)) OR (smartphone)) OR (cell phone)) OR (mobile technology)) OR (mobile device)) OR (mobile-based phone)) OR (tablet computer)) OR (IPAD)) OR (pda)) OR (mhealth application))) AND (crowdsensing)	33
4	Search: (((((((((((((((mhealth) OR (telemedicine)) OR (wireless technology)) OR (mobile phone)) OR (smartphone)) OR (cellphone)) OR (mobile technology)) OR (mobile device)) OR (mobile-based phone)) OR (tablet computer)) OR (IPAD)) OR (pda)) OR (mhealth application))) AND (crowdsensing)) AND (disease)	1

**^a^**Filters include publications from January 1, 1945, to December 31, 2019.

### Study Selection

TT will retrieve all relevant articles from various databases, based on the finalized search strategy. All the literature obtained will be saved in Endnote reference management software (Clarivate Analytics). Both authors will independently screen the titles and abstracts of retrieved studies for eligibility. Both authors will make a final assessment for inclusion using the full-text article. Discrepancies and disagreements will be resolved by both authors.

### Data Extraction

A standardized data extraction form adapted from a study by Tokosi et al [30] will be used for data extraction. Full texts of selected abstracts will be retrieved and data extracted in line with the prespecified template. The key information to be extracted includes the following: author name(s) and year of the study, type of participant/study population and demographic characteristics; type of mHealth device used (eg, mobile phones, PDAs, smartphones, tablets); type of intervention (eg, SMS, MMS, video, text, audio); nature of the mHealth intervention (eg, awareness, diagnosis, treatment); type of study (eg, randomized, nonrandomized); type of outcome measured; and findings/results.

Following Saidi et al’s [32] procedure, data will be entered into Review Manager software, version 5.3. (Cochrane). Both authors will verify the data entered for missing or incorrect data. If both authors cannot agree on missing data entry, an independent third party will be consulted to mediate. Missing data will be requested from study authors via email [21]. If no response is received from study authors, an attempt will be made to impute missing SD or standard error values using data from other similar studies in the review with similar methods and sample sizes, as recommended by Wiebe et al [33].

### Assessing Risk of Bias

The authors will use recommendations by the International Cochrane Collaboration to independently assess the risk of bias [34]. These criteria include randomization sequence generation, treatment allocation concealment, blinding of participants, incomplete outcome data, selective outcome reporting, and other sources of bias [30]. All included studies will be scored for bias using these criteria. A descriptive summary for each scoring will be recorded. Discrepancies between the review authors regarding the risk of bias in particular studies will be resolved by dialogue, with involvement of an independent third party, where necessary.

### Data Analysis and Synthesis

The extracted data will be presented in an evidence table adapted from Saidi et al’s [32] study. A descriptive synthesis will be undertaken in accordance with the Centre for Reviews and Dissemination [35]. Continuous outcomes will be ascertained by calculating mean differences and SDs. Ratios and their corresponding 95% CIs will be determined for dichotomous outcomes. Heterogeneity will be used to examine participants, interventions, and outcomes of each study. The statistical test for heterogeneity will include the *I*^2^ test which quantifies heterogeneity; this test will allow for the quality of evidence to be validated [34]. Data will be pooled; where collected data are sufficiently similar, a meta-analysis will be conducted. Similarly, where the variability between studies is high, the results will not be pooled and a narrative synthesis will take place [30]. When appropriate, a subgroup analysis will be used to determine if varying mHealth crowdsensing applications have an impact on chronic disease awareness and in what context this occurs. Subgroups to be considered for this analysis will include age grouping and geographical region.

Various sensitivity analyses as espoused by Tokosi et al [30] will be performed, including analysis conducted based on the study quality (risk of bias and level of participant dropout) to investigate possible sources of heterogeneity. Another analysis will be used to determine how excluded studies could have influenced the overall result. A final analysis to determine how the result would differ from other study results should there be only high-quality studies included will be used [34].

## Results

The literature sourcing with regard to the inclusion and exclusion criteria of the study is ongoing. All data to be extracted will be grouped under various headings as specified in the data extraction form, which will include the following: basic study information (eg, author name, year); type of participant/study population and demographic characteristics; type of mHealth device used (eg, mobile phones, PDAs, smartphones, tablets etc); type of intervention (eg, SMS, MMS, video, text, audio); nature of the mHealth intervention (eg, awareness, diagnosis, treatment); type of study (eg, randomized, nonrandomized); type of outcome measured; and findings/results [30]. The preliminary search query found 1 paper**.** The literature search will commence in mid-March 2021 and will end in April 2021. It is anticipated that the final review will be completed in May 2021.

## Discussion

This review will identify and describe the impact of MHCS interventions on chronic diseases. The findings of the systematic review will inform the design of mHealth interventions for chronic diseases. Furthermore, the study will highlight which mHealth technology modalities (eg, SMS) are appropriate for the target audience when creating awareness for chronic diseases.

## Appendix

Multimedia Appendix 1 PRISMA-P (Preferred Reporting Items for Systematic Reviews and Meta-Analysis Protocols) 2015 checklist.

Multimedia Appendix 2 Search query.

## Abbreviations

CDC: Centers for Disease Control and Prevention
COLLEGA: Collaborative Emergency Group Assistant
HHS: Health and Human Services
MHCS: mobile health crowdsensing
mHealth: mobile health
MMS: multimedia message service
PDA: personal digital assistant
PRISMA-P: Preferred Reporting Items for Systematic Reviews and Meta-Analysis Protocols
PROSPERO: The International Prospective Register of Systematic Reviews
