# Supplementary material for: Mobile Health Crowdsensing (MHCS) Intervention on Chronic Disease Awareness: Protocol for a Systematic Review
Source: JMIR Res Protoc. 2021 Mar 19;10(3):e24589. doi: 10.2196/24589 (PMC8088872; doi:10.2196/24589)
Supplement: Multimedia Appendix 2 [file resprot_v10i3e24589_app2.doc]

**APPENDIX 1 –** Preliminary search query classification

| Number | Query | Items found |
| --- | --- | --- |
| #1 | Search: ((((((((((((mhealth) OR (telemedicine)) OR (wireless technology)) OR (mobile phone)) OR (smartphone)) OR (cellphone)) OR (mobile technology)) OR (mobile device)) OR (mobile-based phone)) OR (tablet computer)) OR (IPAD)) OR (pda)) OR (mhealth application) Filters: from 1945/1/1 - 2019/12/31 | [102,787](https://pubmed.ncbi.nlm.nih.gov/?term=((((((((((((mhealth)+OR+(telemedicine))+OR+(wireless+technology))+OR+(mobile+phone))+OR+(smartphone))+OR+(cellphone))+OR+(mobile+technology))+OR+(mobile+device))+OR+(mobile-based+phone))+OR+(tablet+computer))+OR+(IPAD))+OR+(pda))+OR+(mhealth+application)&filter=dates.1945/1/1-2019/12/31&sort=relevance) |
| #2 | Search: (((((((((((((mhealth) OR (telemedicine)) OR (wireless technology)) OR (mobile phone)) OR (smartphone)) OR (cellphone)) OR (mobile technology)) OR (mobile device)) OR (mobile-based phone)) OR (tablet computer)) OR (IPAD)) OR (pda)) OR (mhealth application)) AND (crowdsourcing) Filters: from 1945/1/1 - 2019/12/31 | 162 |
| #3 | Search: ((((((((((((((mhealth) OR (telemedicine)) OR (wireless technology)) OR (mobile phone)) OR (smartphone)) OR (cellphone)) OR (mobile technology)) OR (mobile device)) OR (mobile-based phone)) OR (tablet computer)) OR (IPAD)) OR (pda)) OR (mhealth application))) AND (crowdsensing) Filters: from 1945/1/1 - 2019/12/31 | 33 |
| #4 | Search: (((((((((((((((mhealth) OR (telemedicine)) OR (wireless technology)) OR (mobile phone)) OR (smartphone)) OR (cellphone)) OR (mobile technology)) OR (mobile device)) OR (mobile-based phone)) OR (tablet computer)) OR (IPAD)) OR (pda)) OR (mhealth application))) AND (crowdsensing)) AND (disease) Filters: from 1945/1/1 - 2019/12/31 | 1 |
